# Global analysis of kinetics reveals the role of secondary nucleation in recombinant spider silk self‐assembly

**DOI:** 10.1002/pro.4722

**Published:** 2023-08-01

**Authors:** Veronika Hovanová, Andrej Hovan, Gabriel Žoldák, Erik Sedlák, Martin Humenik

**Affiliations:** ^1^ Center for Interdisciplinary Biosciences, Technology and Innovation Park P.J. Šafárik University Košice Slovakia; ^2^ Department of Biophysics, Faculty of Science P.J. Šafárik University Košice Slovakia; ^3^ Center for Interdisciplinary Biosciences Cassovia New Industry Cluster (CNIC) Košice Slovakia; ^4^ Department of Biochemistry, Faculty of Science P.J. Šafárik University Košice Slovakia; ^5^ Department of Biomaterials, Faculty of Engineering Science University Bayreuth Bayreuth Germany

**Keywords:** fibrils, recombinant protein, secondary nucleation, self‐assembly, spider silk

## Abstract

Recombinant spider silk proteins can be prepared in scalable fermentation processes and have been proven as sources of biomaterials for biomedical and technical applications. Nanofibrils, formed through the self‐assembly of these proteins, possess unique structural and mechanical properties, serving as fundamental building blocks for the fabrication of micro‐ and nanostructured scaffolds. Despite significant progress in utilizing nanofibrils‐based morphologies of recombinant spider silk proteins, a comprehensive understanding of the molecular mechanisms of nanofibrils self‐assembly remains a challenge. Here, a detailed kinetic study of nanofibril formation from a recombinant spider silk protein eADF4(C16) in dependence on the protein concentration, seeding, and temperature is provided. For the global fitting of kinetic data obtained during the fibril formation, we utilized the online platform AmyloFit. Evaluation of the data revealed that the self‐assembly mechanism of recombinant spider silk is dominated by secondary nucleation. Thermodynamic analyses show that both primary and secondary nucleations, as well as the elongation step of the eADF4(C16), are endothermic processes.

## 1 INTRODUCTION

Spider silk, especially *dragline* silk, due to its unusual combination of tensile strength, elasticity, and break resistance as well as biocompatibility (Arakawa et al., 2022; Bourzac, 2015; Fritz, 2000; Liu et al., 2017), has been used as an inspiration for preparation of protein‐based materials (Arndt et al., 2022; Heidebrecht et al., 2015; Xia et al., 2010). However, the cannibalistic behavior of spiders and the limited production of natural silk have led to the establishment of recombinant production of protein variants with natural or engineered sequences in diverse host organisms (Abascal & Regan, 2018; Heidebrecht & Scheibel, 2013; Humenik et al., 2011; Whittall et al., 2021). Beyond the scope of fiber formation, the capability to form biodegradable and biocompatible nanofibrils, microparticles, coatings, foams, and hydrogels in dependence on a simple modification of environmental processing conditions, such as solvents, ionic strength, combinations of cations and anions as well as pH, make recombinant spider silk proteins and their derivatives an attractive material for biomedical applications, tissue engineering and biofabrication (Aigner et al., 2018; Esser et al., 2021; Florczak et al., 2018; Lamberger et al., 2022; Zeplin et al., 2014). One well‐established recombinant variant, known as eADF4(C16), mimics the core domain of fibroin 4 from the dragline silk of the European garden spider, *Araneus diadematus (*Huemmerich et al., 2004; Koeppel et al., 2021). The engineered protein comprises repetitive C‐modules that incorporate key natural motifs, including (Ala)_8_ and GPGXY (Huemmerich et al., 2004; Rammensee et al., 2008).

It has been demonstrated that the self‐assembly of eADF4(C16) into cross‐β fibrils is triggered by the presence of kosmotropic anions, such as phosphates or sulfates, at low concentrations, independently on the cation counterpart (Humenik et al., 2015b) and other physical stress, such as shear forces. The ability of eADF4(C16) to undergo fibril assembly in response to anions is particularly intriguing considering its highly negative charge due to its repetitive nature. Hence, the eADF4(C16) fibril assembly resembles rather a general property of intrinsically unstructured proteins to transform into a thermodynamically stable cross‐β sheet structure, observed for plethora of proteins after unfolding (Eichner & Radford Sheena, 2011). Generally, the formation and growth of amyloid fibrils from intrinsically unstructured proteins involve several critical molecular steps, including primary nucleation and elongation. Primary nucleation serves as the initial step, where monomeric proteins or peptides self‐assemble into oligomers or nuclei, acting as seeds for further fibril growth. Elongation occurs as monomers or smaller aggregates bind to the ends of existing fibrils, extending their structure. Additionally, amyloid fibril formation can also occur through secondary pathways, such as secondary nucleation or fragmentation. Secondary nucleation arises when preexisting fibrils interact with monomers or smaller aggregates, generating new nuclei that facilitate accelerated fibril formation. Fragmentation involves the breakage of existing fibrils into smaller fragments, creating new ends that serve as templates for elongation (Cohen et al., 2012; Meisl et al., 2016; Meisl et al., 2022; Tornquist et al., 2018).

Although the molecular mechanism involved in cross‐β fibril formation is a subject of broad research interest (Ikura et al., 2022; Meisl et al., 2022; Michaels et al., 2018; Sinnige, 2022), there have been limited attempts to elucidate the specific molecular mechanism in case of structural protein forming β‐sheet rich fibrils such as eADF4(C16) (Humenik et al., 2014; Humenik et al., 2015b; Slotta et al., 2007) or fibroin (Knowles & Mezzenga, 2016). Nanofibrils play a crucial role as the fundamental building blocks of hydrogels, significantly influencing their structure, mechanical properties, and functionality. Thus, gaining deeper insights into this process will have significant implications for understanding the properties of materials constructed from nanofibrils of the protein eADF4(C16), such as bulk hydrogels for 3D printing (DeSimone et al., 2017; Lechner et al., 2021; Schacht et al., 2015; Steiner et al., 2021) and nanohydrogels for surface modification and patterning (Heinritz et al., 2022; Humenik et al., 2020; Lamberger et al., 2022). Understanding the self‐assembly of this charged, intrinsically unstructured protein extends beyond the realm of biomaterials development and contributes to the broader understanding of cross‐β fibril formation, which holds relevance in both biomedical and materials science contexts (Buell, 2019; Grigolato & Arosio, 2021; Kamada et al., 2023; Knowles & Mezzenga, 2016; Scollo & La Rosa, 2020; Wei et al., 2017).

Here, we present for the first time a detailed global analysis of the eADF4(C16) cross‐β fibrils formation, using integrated rate law kinetics implemented in the platform AmyloFit (Meisl et al., 2016). The self‐assembly of the protein eADF4(C16) was examined by varying the protein concentration and temperatures in the presence or absence of eADF4(C16) seeds. Through this approach, we were to elucidate that the fibril formation process involves not only primary processes, such as primary nucleation and elongation, but also secondary pathways, including secondary nucleation.

## 2 MATERIALS AND METHODS

All chemicals, except glucose oxidase (GOX), were purchased from Carl Roth (Germany). GOX was obtained from Merck. Ultrapure water from a Millipore system (Merck KGaA, Germany) was used in the experiments.

### 2.1 Proteins solubilization

The recombinant spider silk protein eADF4(C16), containing the 16‐times repetitive C‐module with amino acid sequences: GSSAAAAAAAASGPGGYGPENQGPSGPGGYGPGGP was produced and purified as previously reported (Huemmerich et al., 2004). Purity and identity of the protein was determined using Matrix‐assisted laser desorption time‐of‐flight (MALDI‐TOF) spectrometry (Figure S1). Before use in kinetic assays, the protein was dissolved in guanidinium thiocyanate solution (6 M) and dialyzed against 10 mM Tris/HCl, pH 8.0 at room temperature. The buffer was changed four times every 2.5 h and one time over night. To obtain a monomeric protein state without aggregates and oligomers, the dialyzed samples were centrifuged in an ultracentrifuge (Optima MAX‐XP, Beckman‐Coulter, USA) at 185,000 *g* at 4°C for 50 min, and the concentration of soluble protein was determined using a UV–VIS spectrophotometer (NanoDrop 1000, Thermo Fisher, USA). The reliability of the procedure has previously been demonstrated using a size exclusion chromatography coupled to light scattering detector showing neat monomer in the solution (Humenik & Scheibel, 2014).

### 2.2 MALDI‐TOF mass spectrometry

Dialyzed protein was desalted using a protocol for C4‐ZipTips® and co‐eluted with a matrix solution (60% [v/v] Acetonitrile, 0.1% [v/v] TFA, 20 mg/mL sinapinic acid). The sample (0.5 μL) was then applied onto a stainless‐steel target plate, dried and analyzed in a Bruker Autoflex mass spectrometer. The spectra were evaluated with mMass software version 5.5.0.

### 2.3 Kinetic assays at quiescent conditions

The protein solution after ultracentrifugation was used to prepare a series of samples with a concentration between 5 and 40 μM. Assays were initiated by the addition of phosphate buffer (KPi), pH 8.0, in final concentration 150 mM, to the protein solutions, resulting in a change of turbidity at 340 nm, recorded on the spectrophotometer (*Varian Cary 50* UV–Vis Spectrophotometer, Germany) at 20–26°C and in the 96‐well plate reader (SpectraMax iD5 Molecular Devices, USA) without agitation at 30–40°C every 10 min.

### 2.4 Fluorescence measurement of ThT and ANS

Thioflavin T (ThT) and 1‐anilino‐8‐naphthalene sulfonate acid (ANS) were dissolved in MQ water, filtered through a 0.2 mm syringe filter and concentrations were calculated from the absorbance measured at 416 nm for ThT and 351 nm for ANS in a UV–visible spectrophotometer (Cary 50 UV, Varian, Germany) using molar extinction coefficients: 36000 M^−1^·cm^−1^ for ThT and 5200 M^−1^·cm^−1^ for ANS (Groenning et al., 2007; Ziaunys et al., 2019). On a plate‐reader (SpectraMax iD5 Molecular Devices, USA) was recorded change of fluorescence of 1 mM ANS (355 nm, 460 nm) and 30 μM Thioflavin‐T (460 nm, 535 nm) with 10 μM protein solution and 150 mM KPi, pH 8.0, at 30°C every 10 min.

Individual ANS spectra were measured at a protein concentration of 10 μM with 150 mM Pi and 1 mM ANS (20 mm stock dissolved in 70% ethanol) following excitation at 395 nm. Fluorescence spectra were measured both before and after 24 h of incubation at 30°C. Fluorescence measurements of individual ANS spectra were performed on spectrofluorimeter Shimadzu RF‐5000.

To assess hydrophobic patches on eADF4(C16) and/or amorphous aggregates formed during the protein fibrillization, we utilized ANS (8‐anilinonaphtalene‐1‐sulfonic acid) induced fluorescence upon binding to hydrophobic region (Gasymov & Glasgow, 2007; Zoldak et al., 2004). Molten globule state of GOX from *Aspergillus niger* formed after re‐cooling of the protein from 80°C as a result of irreversible thermal denaturation has been used as a reference to demonstrate such changes in ANS fluorescence (Zoldak et al., 2004). We compared ANS fluorescence in 5 μM of GOX (80 kDa/monomer) upon thermal denaturation with 10 μM eADF4(C16) in monomeric and fibrillar states.

### 2.5 Preformed seeds

Mature fibrils (assembled from 20 μM eADF4(C16) in 150 mM KPi at room temperature for 48 h) were sonicated using an ultrasonic homogenizer (Sonopuls HD 3200, Germany, MS73 tip set, 10% amplitude) for 15 s with six repetitions while keeping the samples on ice. The seeds were added at 0.5% or 30% (w/w, seed/soluble protein) to protein solutions in the presence of 150 mM KPi, and then the change of turbidity over time was recorded.

### 2.6 Shear‐induced aggregation

eADF4(C16) protein solution (15 μM in 150 mM KPi) was incubated in orbital shaker at 22 rpm at 20°C for 24 h. The resulting macroscopic aggregates were visualized using a bright field microscopy and cross polarized light.

### 2.7 Curve fitting with AmyloFit

Kinetic datasets of the protein eADF4(C16) (10–60 μM) in the presence of 150 mM kosmotropic phosphate salt, with or without seeds, were fitted using the online platform AmyloFit (www.amylofit.ch.cam.ac.uk) (Meisl et al., 2016). The entire analysis was conducted following the protocol of Meisl et al. (2016). A detailed description of the analysis can be found in the Supporting Information.

### 2.8 Transmission electron microscopy

The protein samples (10 μL) were deposited on Pioloform‐carbon‐coated 200‐mesh copper grids (Plano GmBH, Germany), and incubated for 1 min at room temperature. Next, the grid was washed with 5 μL of water and negatively stained with 2% uranyl acetate for 1 min. Images were recorded using a Zeiss LEO EM922 Omega microscope (Zeiss Microscopy, Jena, Germany), which was operated at 200 kV accelerating voltage. Images were recorded by a bottom‐mounted CCD camera system (Ultrascan 1000, Gatan, München, Germany) and processed with a digital imaging processing system (Digital Micrograph GMS 1.9, Gatan, München, Germany).

### 2.9 Atomic force microscopy

Fibrils assembled from 15 μM eADF4(C16) in 150 mM KPi, pH 8.0 for 48 h were deposited on a freshly cleaved mica substrate, incubated for 5 min, and washed four times with water. The surface scans were performed on a Dimension ICON with Nanoscope V controller in TappingMode™ using Si cantilevers (OTESPA‐R3, resonance frequency of 300 kHz, spring constant of 26 N/m; Bruker, USA). Data were processed using a Gwyddion‐2.62. software.

### 2.10 Circular dichroism spectroscopy

The far‐UV circular dichroism spectroscopy (CD) spectra were performed using a spectropolarimeter (JASCO J‐815, Japan) equipped with a Peltier cuvette holder at a temperature of 20°C. The protein samples were diluted with water to achieve a final concentration of 5 μM. Scans were acquired by averaging three individual spectra in the range of 190–250 nm, with data points collected every 1 nm.

## 3 RESULTS

### 3.1 Global analysis of eADF4(C16) aggregation at different concentrations

The self‐assembly of the purified protein eADF4(C16) (Figure S1) was investigated by monitoring turbidity kinetics at 340 nm and ThT fluorescence in the presence of 150 mM KPi at pH 8.0 (Figure S2a). ThT is a commonly used fluorescent dye for monitoring the formation of cross‐β sheet fibrils, due to significantly increased fluorescence upon binding to the fibrils (Linse, 2021; Naiki et al., 1989; Sulatsky et al., 2020). Another frequently employed technique for measuring fibrillization is the change of turbidity (Linse, 2021; Zhao et al., 2016), which has also been used in the study of eADF4(C16) self‐assembly (Humenik et al., 2014; Humenik et al., 2015b; Neubauer et al., 2021; Schacht & Scheibel, 2011). Previously, we have shown that the increasing turbidity signal correlated with the rising fraction of insoluble fibrils formed by different length variants of eADF4(Cn) (Humenik et al., 2014). Here, the similarity of the ThT signals suggested the possibility of investigating eADF4(C16) self‐assembly using turbidity changes. ANS serves as a fluorescent probe for binding to hydrophobic sites on proteins (Zoldak et al., 2004). ANS fluorescence upon binding to hydrophobic patches on protein surface or to amorphous aggregates is accompanied by the blue shift from ~520 nm to ~465 nm and significant increase in the fluorescence intensity. An addition of the monomeric form to ANS led to only a small increase in the fluorescence intensity of ANS without significant shift in the fluorescence maximum position (Figure S2b). On the other hand, an addition of the eADF4(C16) fibrils is accompanied by slight blue shift to ~482 nm and ~ 3‐fold increase in the ANS fluorescence intensity. Comparison with the ANS fluorescence bound to GOX molten globule state with the fluorescence maximum position at ~472 nm and significantly increased fluorescence intensity suggests the absence of binding sites on the monomeric form and only slight increase of ANS binding to eADF4(C16) fibrils. Although, our results cannot exclude formation of amorphous aggregates in parallel to fibrillization of eADF4(C16), an absence of correlation in fibrillization kinetics of the protein suggest that a formation of fibrils and a potential amorphous aggregates are independent processes.

**FIGURE 1:**
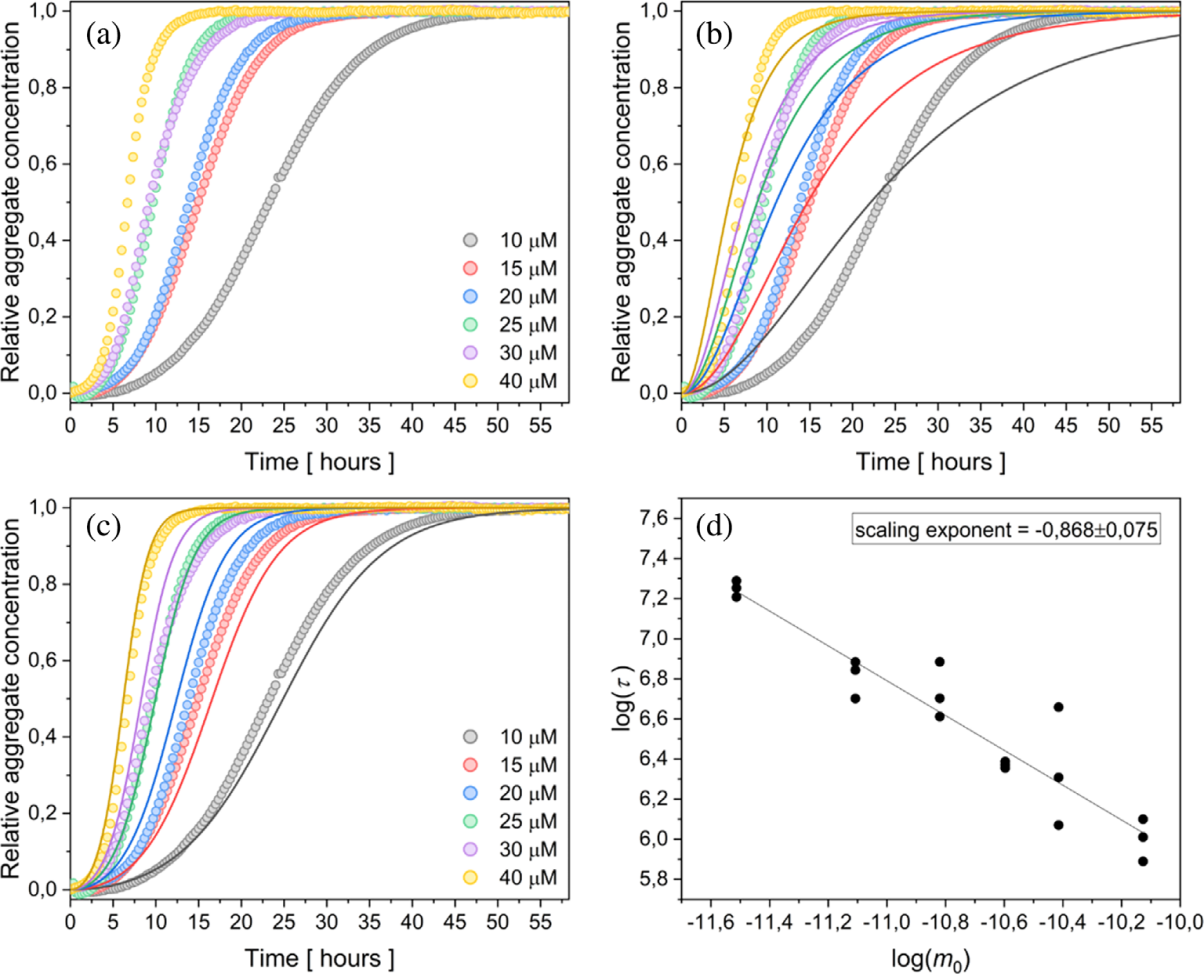
Self‐ assembly kinetics of eADF4(C16) protein in the presence of 150 mM KPi, at 20°C. (a) Normalized changes in turbidity at 340 nm upon formation of fibrils from the monomeric protein in the concentrations range 10–40 μM. (b) Data fitting the nucleation‐elongation model. The global fit function did not adequately describe the kinetic. c) Data fitting with the global model including the secondary nucleation revealed a higher accuracy to (b). d) Power law dependence of half‐times on protein concentration. The filled area corresponds to standard deviation of the data. In some cases, the standard deviation is smaller than a symbol size.

Protein eADF4(C16) in the monomeric form was examined at various concentrations (10–40 μM), in the presence of 150 mM KPi. Due to the demanding reproducibility of the data, the initial conditions (buffer properties such as pH, ionic strength, and temperature) were strictly defined and maintained. Hence, recombinant protein eADF4(C16) was used after ultracentrifugation, which have been shown previously to remove oligomers and aggregates after dialysis (Humenik et al., 2015a; Humenik et al., 2015b). The kinetics of protein aggregation at six different protein concentrations (Figure 1) showed that the overall fibrillization process accelerated with the increasing protein concentration. To establish a connection between experimental observations and microscopic processes, the AmyloFit web platform (Meisl et al., 2016) was employed. This platform enables the determination of the dominant mechanism of aggregation through global fitting. Following the published protocol (Meisl et al., 2016), the half‐time *τ* was plotted against the initial protein concentration (*m*_0_) (Figure 1d). The linear dependence of the log(τ) versus log(*m*_0_) implied that only one aggregation mechanism is dominant in the whole process. The scaling exponent, with a value of −0.868 ± 0.075, suggested two possible models to describe protein aggregation, namely: nucleation‐elongation (Figure 1b) and secondary nucleation (Figure 1c).

The simplest nucleation‐elongation model did not fit the measured data satisfactorily (Figure 1b) therefore it was necessary to consider the models that include secondary pathways. Based on the fitting of the data with the secondary nucleation model (Figure 1c), it was presumed that this process plays an important role in the fibril formation from eADF4(C16). In addition, the data have been fitted with fragmentation‐dominated and fragmentation with secondary nucleation models to prevent misinterpretation of the data. Although the fits provided satisfactory results (Figure S3), the shape of the transitions from lag phase and saturation phase were sharper and did not reproduce the curve shapes as nicely as when considering secondary nucleation alone. Moreover, the fragmentation modes would be more plausible in case of agitation conditions, however, herein the quiescent self‐assembly was studied.

**FIGURE 2:**
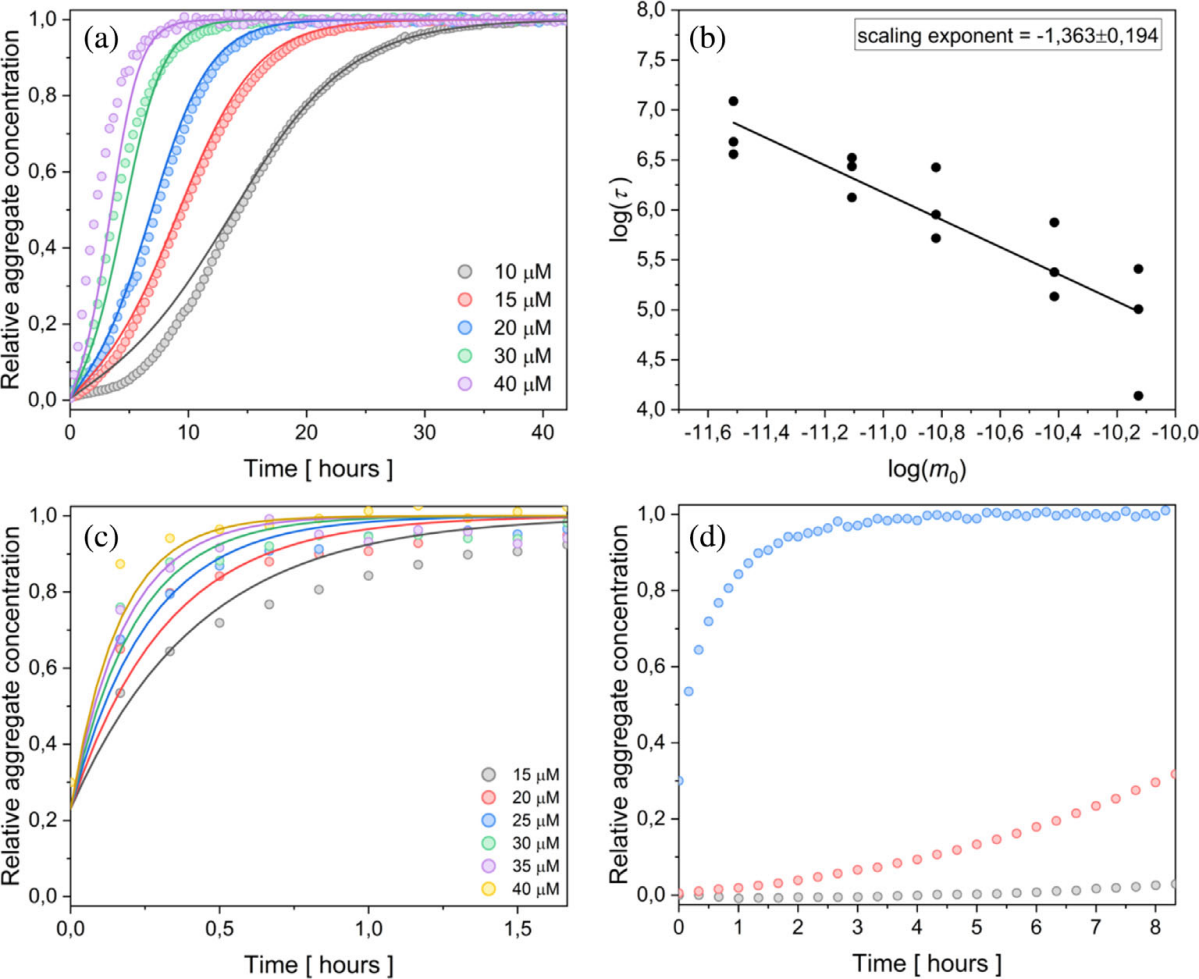
Self‐assembly of the eADF4(C16) in 150 mM KPi, pH 8.0 in the presence of nucleation seeds at 20°C. (a) Self‐assembly in presence of seeds (0.5% w/w of the initial protein concentration) as analyzed by global fit including secondary nucleation. (b) Corresponding power law dependence of half‐times as a function on the protein concentration. (c) Kinetic data set after addition of 30% w/w seeds. (d) Comparison of the self‐assembly kinetics at 15 μM protein in absence (gray), and upon addition of 0.5% (red) and 30% (blue) of seeds.

The addition of preformed seeds in a small amount can serve as a qualitative prove of secondary nucleation presence in the assembly processes (Meisl et al., 2016). The protein concentration range of 10–40 μM and the addition of 0.5% w/w seeds in the presence of 150 mM KPi were used for verification of chosen kinetic model, and the obtained data were fitted with the same secondary nucleation model. The global fit of the given model described the course of the curves well (Figure 2a). The correctness of the chosen model was confirmed by the acceleration of the process after addition of seeds (0.5% w/w) (compare the time scales in Figures 1c and 2a), as well as by fitting the data with the same model of secondary nucleation for both, the unseeded and the seeded assembly. Plotting the half‐times on a log–log graph against the concentration of protein monomers once again revealed a linear function (Figure 1d vs. Figure 2b), confirming the dominant involvement of the secondary nucleation mechanism.

The kinetic data were obtained at different protein concentrations upon addition of 30% w/w seeds (Figure 2c) as well. The secondary nucleation model correctly described the experimental data, despite the incompleteness of the curves. Due to the high concentration of seeds, the signal corresponding to fibril formation was captured after the half‐time of the fibrillization. This is clearly demonstrated by comparing the fibrillization rates (in the first 8 h) for 15 μM protein eADF4(C16) protein in the presence of 150 mM KPi in the absence (gray) and presence of 0.5% (red) and 30% (blue) nucleation seeds (Figure 2d).

### 3.2 Morphology of eADF4(C16) fibrils

**FIGURE 3:**
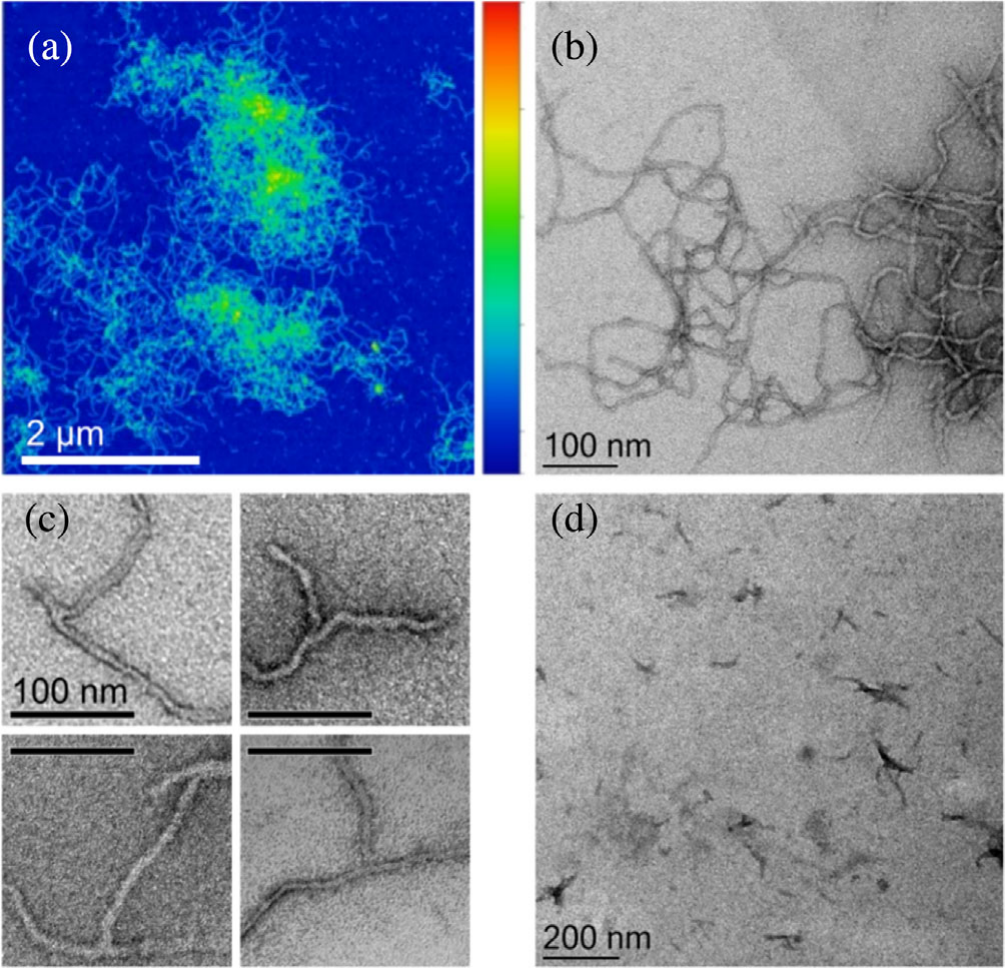
Morphology of eADF4(C16) fibrils, prepared by addition 150 mM KPi to 15 μM eADF4(C16) after incubation for 48 h at 20°C as corresponds to the experiment in Figure 1, was visualized using atomic force microscopy (AFM) (heights) in (a) and transmission electron microscopy (TEM) in (b) and (c). In (c) focus on fibril branching is shown. (d) TEM image of seeds formed after the sonication of mature fibrils. Color bar in (a) represents heights between 0 nm (deep blue) and 22 nm (deep red).

The protein fibrous structures, formed in the presence of KPi, were imaged using AFM and TEM after reaching the stationary phase (Figure 3a, b). A closer examination of selected fibrils (Figure 3c) revealed branching, confirming the involvement of the secondary nucleation mechanism in fibril formation. Figure 3d shows freshly prepared nucleation seeds that were added to the systems to verify the chosen model.

### 3.3 Global fitting analysis of eADF4(C16) self‐assembly at different concentrations and temperatures

To gain a deeper understanding of the molecular steps in the aggregation of the recombinant spider silk protein eADF4(C16), several measurements were performed at various protein concentrations and temperatures 20, 23, 26, 30, 33, 36, and 40°C. To investigate the potential impact of temperature on the secondary structure of the formed aggregates, CD spectra were measured. The obtained results consistently revealed the presence of β‐sheets in all tested cases, suggesting that the nanofibril structure remains unchanged with increasing temperatures (Figure S4a).

**FIGURE 4:**
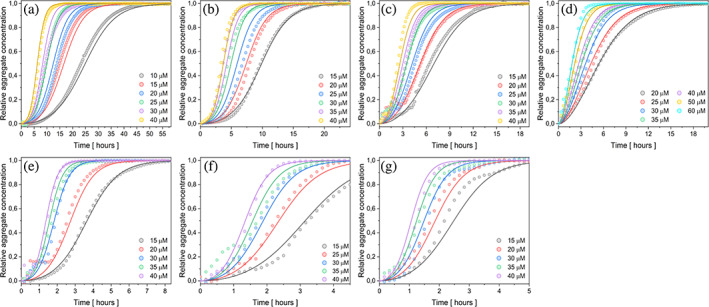
Self‐assembly kinetics of eADF4(C16) in the presence of 150 mM KPi, pH 8, at 20°C (a), 23°C (b), 26°C (c), 30°C (d), 33°C (e), 36°C (f), and 40°C (g). Data represent average of three replicates for each condition. The global fitting of all data was done using AmyloFit. The best fit was obtained using the secondary nucleation model for all series. Note the different scales on the time axes. For better visualization of standard deviation of the data, the above curves are shown with an offset in Figure S5.

The evaluation of data sets using the AmyloFit platform (Meisl et al., 2016) revealed that an increase in temperature accelerates the process of fibril formation (Figure 4; Figure S4b). The log–log plots of the half‐times versus monomer concentrations showed a linear dependence with a negative scaling exponent of −0.947 ± 0.146 (Figure S6), indicating the same secondary nucleation mechanism in all studied cases. Based on the protocol (Meisl et al., 2016), the value of *n*_2_ was estimated to be 1 from the equation γ ≈ −(*n*_2_ + 1)/2. Therefore, in the fitting procedure the value of *n*_2_ was set to 1. Additionally, the value of *n*_c_ was set to 2, which is a recommended value in the AmyloFit manual (https://amylofit.com/static/fitter_manual.pdf) and is commonly used (Cohen et al., 2013; Meisl et al., 2014). The chosen model correctly describes the course of measured kinetics with these fixed parameters. In fact, even when these parameters were set free, the fits converged to similar values close to 1, and 2 for *n*_2_, and *n*_c_, respectively.

**FIGURE 5:**
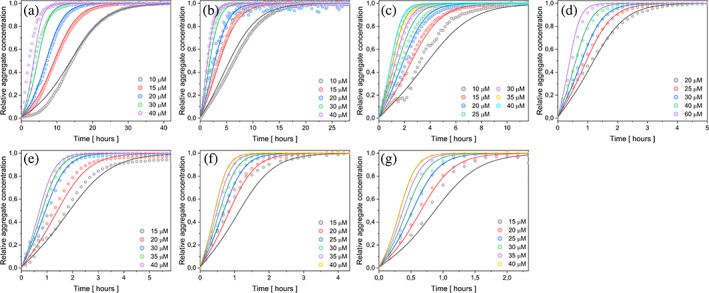
Seeded self‐assembly of eADF4(C16) in the presence of 150 mM KPi, pH 8 and 0.5% w/w of preformed seeds at 20°C (a), 23°C (b), 26°C (c), 30°C (d), 33°C (e), 36°C (f), and 40°C (g). The data represent an average of three replicates. The global fitting was performed using the secondary nucleation model. Note the different scales on the time axes. For better visualization of standard deviation of the data, the above curves are shown with an offset in Figure S7.

To verify the mechanism for each temperature set, we performed a series of self‐assembly kinetics experiments by introducing a small quantity of nucleation seeds (0.5% w/w) (Figure 5). The observed reduction or even absence of the lag phase served as qualitative proof for the involvement of secondary nucleation processes (Meisl et al., 2016), and the log–log plots (Figure S8) with linear dependence further supported our findings. Since the addition of seeds eliminated lag phase at higher temperatures, the differences between individual half‐times were shortened resulting in lower negative values for the scaling exponents.

**FIGURE 6:**
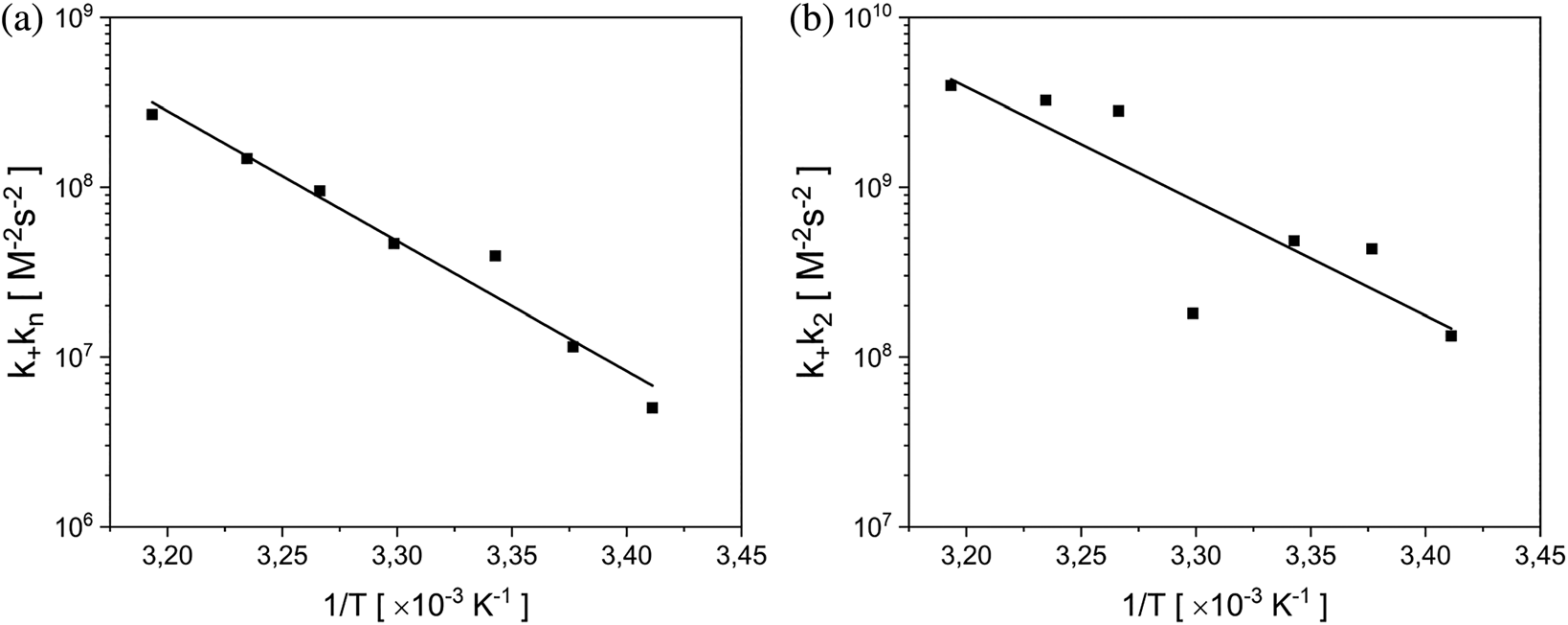
Arrhenius plot of the combined rate parameter for primary (*k*_+_ *k*_n_) in (a) and secondary (*k*_+_ *k*_2_) nucleation in (b) in the self‐assembly of eADF4(C16). The plots show the inversed temperature dependence of the rate parameters in logarithmic scale, as determined from the fits shown in Figure 4.

To connect these experimental observations with the underlying microscopic processes, we applied an analytical approach based on the integral rate law (Cohen et al., 2011; Cohen et al., 2012; Cohen et al., 2013; Cohen et al., 2018). This approach allowed us to determine the values of the rate constants controlling the reaction at each temperature. Although there are distinct microscopic rate constants for primary nucleation (*k*_*n*_), elongation (*k*_+_), and secondary nucleation (*k*_2_), the integrated rate law for protein aggregation revealed that the macroscopic reaction profiles for reactions beginning from monomeric protein, are controlled by only two combinations of these rate constants, *k*_+_ *k*_n_ and *k*_+_ *k*_2_. Using these two kinetic parameters fixed globally across the entire dataset enabled to properly fit all the data across multiple protein concentrations (Figure 4). The global analyses shown in Figure 6 provided values for the combined rate parameters *k*_+_ *k*_n_ and *k*_+_ *k*_2_ at different temperatures. The absence of parameter errors is due to the convergence to the same value during fitting procedure.

**FIGURE 7:**
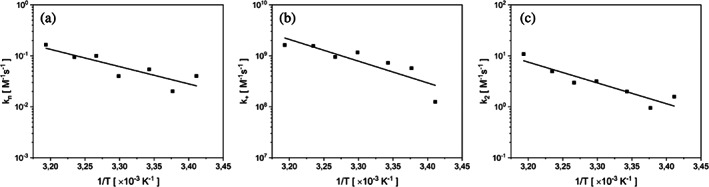
Arrhenius plots of rate constants of (a) primary nucleation, (b) elongation, and (c) secondary nucleation in logarithmic scale. Values of the rate constants were obtained from fits shown in Figure 5.

To calculate the values of the individual rate constants from the combined rate parameters *k*_+_ *k*_n_ and *k*_+_ *k*_2_, the self‐assembly experiments from solutions containing not only protein monomers but also preformed seeds were necessary to perform. The individual parameters, namely (1) *k*_+_—rate constant for elongation, (2) *k*_2_—rate constant for secondary nucleation, were obtained for each temperature using the AmyloFit (Meisl et al., 2016), and are shown in Figure 7b,c. The rate constant for primary nucleation *k*_n_ obtained directly this way was relatively small, around 10^−9^ M^−1^ s^−1^, compared to other rate constants. This is likely due to the lack of lag phase in the early stages of the reaction. The better way to determine *k*_n_ is to divide combined parameter *k*_+_ *k*_n_ obtained from the unseeded dataset by the *k*_+_ values obtained from the seeded experiments. The resulting values of *k*_n_ are shown in Figure 7a. Using a similar approach to obtain *k*_2_ from the combined parameter *k*_+_ *k*_2_ from unseeded experiments, we obtain qualitatively similar results to those obtained from the seeded experiments (Figure S9).

The temperature dependence of the rate constants (here *k*_+_, *k*_n_ and *k*_2_) is described by Kramers theory (Kramers, 1940), in the form of an Arrhenius‐like equation, which links a kinetic rate constant to the free‐energy barrier, ΔG*, in this case the initial state:

(1) $$k=\text{Aexp}\left(-\frac{∆G^{*}}{\mathrm{RT}}\right),$$

for a rate constant *k*, prefactor A, the universal gas constant *R*, and a temperature *T*. According to Cohen et al. (2018):

(2) $$k_{1}k_{2}=A_{1}A_{2}\exp\left(-\frac{{∆G}_{1}^{*}+{∆G}_{2}^{*}}{\mathrm{RT}}\right).$$

The free‐energy barrier ΔG* is also possible to calculate via equation: ΔG* = ΔH*−*T*ΔS*. Based on the gradient of the Arrhenius plots shown in Figure 7, according to ∂(ln *k*)/∂(1/*T*) = −ΔH*/*R*, it was possible to calculate the activation enthalpy, Δ*H*, values of the individual events: primary nucleation, ΔH* = +28.2 ± 7 kJ/mol, elongation, ΔH* = +35.5 ± 9 kJ/mol, and secondary nucleation, ΔH* = +33.9 ± 6 kJ/mol. It is evident from Figure 7 that all rate constants increase as the temperature rises.

*According to the published approach* (Cohen et al., 2018), the entropy of the molecular steps can be identified when the diffusion constant and the radius of the effective reaction volume are known. However, in the case of the protein eADF4(C16), these values are not known, therefore it was not possible to set the entropy associated with the molecular steps of aggregation.

## 4 DISCUSSION

Detailed kinetic analysis of the eADF4(C16) self‐assembly utilizing the integrated rate law as a part of the AmyloFit platform (Meisl et al., 2016) revealed that the fibril formation is dominated by a secondary nucleation mechanism, that is, besides the typical primary pathway from monomer's nucleation to the fibril growth also significant secondary nucleation pathway are present. We presume that during this step, the interaction of monomers with the fibril surface can lead to the formation of new secondary nuclei and subsequent fibril branching. This assumption is in good agreement with the study of Humenik et al. (2015b), where the eADF4(C16) microparticles enable the docking of protein monomers, triggering fibril formation. Similar mechanism has been observed in the growth of fibrils on coatings made of the same protein, resulting in the formation of fibrillar networks with nanohydrogel properties (Humenik et al., 2020). However, the occurrence of branched fibrils was relatively low (Figure 3c) as one could expect from the important role of the secondary nucleation model to fit correctly the self‐assembly kinetics (Figures 1 and 2). There are several possible explanations of this discrepancy: (1) The rate constant for elongation is orders of magnitude higher than that of secondary nucleation what is in accordance with the observation that the eADF4(C16) fibrils are long and only slightly branched. (2) An alternative hypothesis is a detachment of secondary nuclei from the fibril (hydrophobic) surface, a concept that has been discussed in other studies (Cohen et al., 2013; Tornquist et al., 2018). Although, ANS fluorescence indicates hydrophobic patches on the eADF4(C16) fibrils, the ANS fluorescence characteristics (Figure S2b) suggest their relatively low hydrophobicity and thus likely low stability of interaction of secondary nuclei with the primary fibrils. (3) The reduced observation frequency of branched fibrils in TEM or AFM may be explained by the formation of highly entangled fibril bundles (Figure 3a,b), which make it challenging to visually distinguish between branching and overlapping.

The following reasons point on the secondary nucleation as the dominant secondary pathway: (1) significantly better global fit of kinetic data when the secondary nucleation was incorporated (Figure 1), (2) the seed‐induced acceleration of the protein fibrillization (Figure 2), (3) observation of the branched fibrils through TEM imaging (Figure 3), and (4) the absence of curvature in the log–log plots in Figures 1d, 2b, S6, and S8, which supports the involvement of a single dominant mechanism in the amyloid fibril formation (Cohen et al., 2012; Cohen et al., 2013).

However, changing the experimental conditions can result in different dominant mechanism (Sinnige, 2022). Many experimental studies on cross‐β fibril formation, due to protein misfolding in solution, were performed under conditions of mechanical agitation, which supports the fibril fragmentation (Cohen et al., 2013; Furukawa et al., 2008; Hill et al., 2006; Knowles et al., 2009; Lang et al., 2012; Xue et al., 2008). Interestingly, the recent study on regenerated silk fibroin (RSF) demonstrated self‐assembly through the dominant mechanism of secondary nucleation, with shear flow playing a crucial role in inducing nuclei formation and accelerating the process (Kamada et al., 2023). It is important to note that such physical stresses have been observed in the self‐assembly of unstructured proteins, which exhibit conformational properties similar to the recombinant protein eADF4(C16). Therefore, it is reasonable to expect that in addition to the quiescent conditions used in our research, mechanical stress such as shaking or shear flow during fibrillization could further emphasize the dominant role of secondary mechanisms, including fragmentation and secondary nucleation. However, in the present study, we had to exclude mechanical agitation due to the formation of macroscopic aggregates composed of aligned fibrils when eADF4(C16) was subjected to agitation‐induced shear forces. This was visualized by the birefringence using cross‐polarized light microscopy (Figure S10).

The global fitting analysis of the eADF4(C16) self‐assembly at different temperatures allowed us to determine the enthalpy contribution of the free‐energy barrier of each step (primary nucleation, elongation, and secondary nucleation) involved in the fibril formation. The presence of the lag phase implied, in consensus with the generally accepted model of cross‐β fibril formations (Arosio et al., 2015; Cohen et al., 2012; Linse, 2019), that the primary nucleation step is the rate‐determining step with a higher free‐energy barrier. The obtained values of ΔH* for each process are very similar, emphasizing the need for entropy values, which unfortunately we were unable to determine, for the complete thermodynamic description. The positive enthalpy values of the energy barrier for these processes suggest an increasing rate of the reaction with temperature, which imply the role of hydrophobic effect in these steps (Privalov & Gill, 1988). This conclusion agrees with the hydrophobic nature of the poly‐(Ala) motifs present in the repetitive C‐modules of eADF4(C16), which are involvement in the formation of β‐sheets in the recombinant variant (Humenik et al., 2014) as well as in natural spider silk proteins (Humenik et al., 2011). In fact, the increasing number of repetitive modules in the eADF4(Cn) variants resulted in a significant reduction of the lag phase of fibrillization, further supporting the involvement of the hydrophobic effect in primary nucleation (Humenik et al., 2014). It is also important to note that the elongation rate constant is several orders higher than rate constants of primary or secondary nucleation. Nevertheless, it is essential to include process of secondary nucleation for a correct interpretation of the kinetic data for self‐assembly of protein eADF4(C16).

**SCHEME 1:**
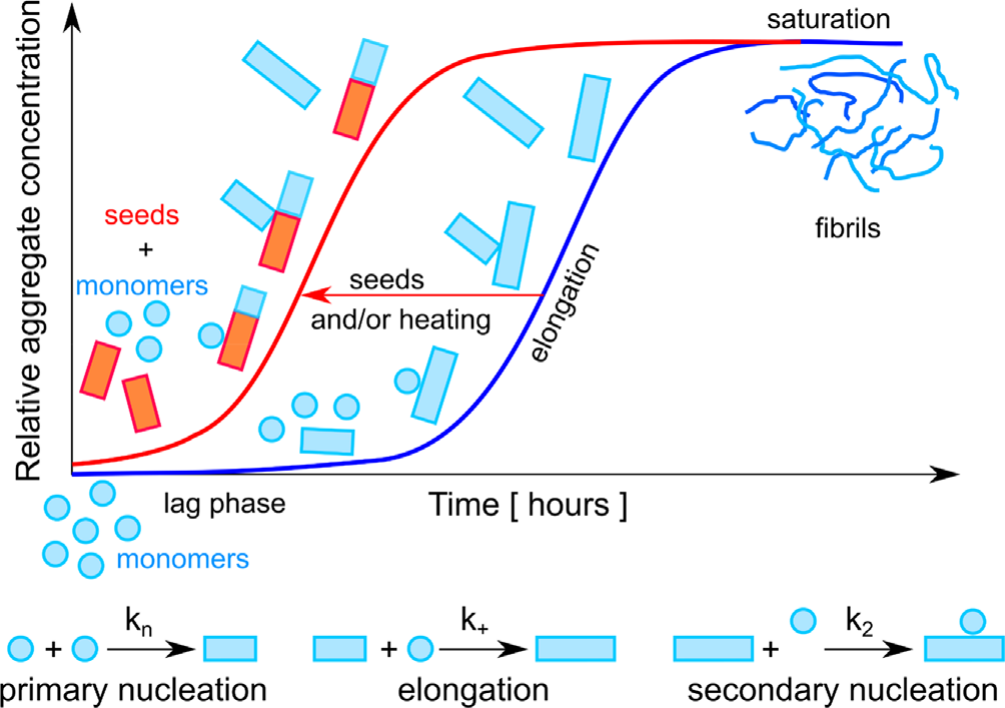
Schematic overview of presented research illustrates the basic mechanism of secondary nucleation and the effect of seeds addition and temperature on the fibrillization of the protein eADF4(C16). Self‐assembly of the recombinant spider silk protein eADF4(C16) was studied by change of turbidity over time. Analysis of the experimental data showed that fibrillization of the protein is governed by a secondary nucleation mechanism. The lag phase is the initial stage, where monomers undergo oligomerization and form small aggregates called primary nuclei. Once the nuclei are formed, elongation takes place, where monomers add to the growing fibril structure, leading to its extension. This phase is characterized by exponential growth of kinetic curve. In additional to primary nucleation and elongation, secondary nucleation plays significant role in accelerating the fibril formation. During secondary nucleation, monomers interact with the surface of existing fibrils, leading, to the formation of secondary nuclei. This process results in the branching of fibrils. Based on the measured kinetic data the individual rate constants for each displayed process were determined. The addition of seeds and increase of temperature accelerate the fibril formation of the protein eADF4(C16).

Importantly, in the category of structural proteins such as here presented eADF4(C16) or recently studied RSF (Kamada et al., 2023) as well as in the category of disease associated peptides/proteins such as Aβ‐variants (Braun et al., 2022; Cohen et al., 2018)_,_ Huntingtin fragments with expanded polyglutamine (Wagner et al., 2018), tau protein (Camargo, Sileikis, et al., 2021), α‐synuclein at mildly acidic pH (Gaspar et al., 2017), as well as islet amyloid polypeptide (Camargo, Chia, et al., 2021; Elenbaas et al., 2022) the self‐assembly into fibrils proceeded via the dominated secondary nucleation mechanism (Scheme 1). These examples imply that plastic surfaces of unstructured proteins result in more frequent involvement of the secondary nucleation mechanism in the corresponding self‐assembly. However, there are few exemptions represented by sickle hemoglobin (Ferrone et al., 1985), carbonic anhydrase (Garg & Kundu, 2016) and insulin (Fodera et al., 2008), which despite being proteins with a rigid structure formed fibrils via the secondary nucleation mechanism. In fact, in very recent work by Meisl et al. (2022), the secondary nucleation has been indicated as the main mechanism of self‐replication under quiescent conditions in many amyloid forming protein. The authors further pointed out that a self‐replication of functional assemblies with a structural role is often slow with the autocatalytic step represented by a secondary mechanism (fragmentation, secondary nucleation) “under control.” In contrast, the fast secondary mechanism could lead to a complex conversion between amorphous and amyloid aggregate morphologies often connected with disease‐associated proteins. Examples of such behavior are represented by Titin I_27_ (Borgia et al., 2013), β_2_‐microglobulin (pH 2.0, higher concentration of NaCl, stirring) (Adachi et al., 2015), prion protein (Lundberg et al., 1997), and non‐amyloidogenic IgE *λ* light chain dimer from human mammalian cells U266 (Arosio et al., 2012). The presence of amorphous aggregates can lead to incorrect data evaluation and molecular mechanism determination (Hall et al., 2015; Zhao et al., 2016). For completion, it is necessary to mention that even the large set of high‐quality data on fibrillization, such as provided on Sup35NM protein in the study of Sharma et al. (2021), was not sufficient to unambiguously determine the dominant self‐assembly mechanism of the protein.

## 5 CONCLUSIONS

In conclusion, using the global fitting of kinetic models incorporated in online platform AmyloFit we could show that the recombinant spider silk protein eADF4(C16) self‐assembles into fibrils via mechanism involving primary and secondary nucleation. As the rate constant for elongation is orders of magnitude higher than the rate constant for primary or secondary nucleation, this provides explanation why we observed long and only slightly branched fibrils. Detailed description of the self‐assembly mechanism of eADF4(C16) opens space for the modulation of the individual rate constants on the level of protein and/or solvent engineering and thus structural properties of the formed fibrils. Hence, in the future studies, it would be beneficial to investigate whether other kosmotropic salts also induce the fibril formation from eADF4(C16) monomers either under the same mechanism or impact more the rates of the secondary nucleation. In such case it could be expected that the resulting fibrils would be more branched. This understanding will be valuable in the development of fibril‐based hydrogels from eADF4(C16) with anticipated higher elastic moduli, thereby influencing for example fidelity of 3D printing or even cell differentiation in biofabrication procedures.

## AUTHOR CONTRIBUTIONS

**Veronika Hovanová**: Investigation; formal analysis; writing—original draft. **Andrej Hovan**: Investigation; formal analysis; writing—original draft. **Gabriel Žoldák**: Conceptualization; formal analysis; funding acquisition. **Erik Sedlák**: Conceptualization; formal analysis; supervision; funding acquisition; writing—review & editing. **Martin Humenik**: Conceptualization; formal analysis; funding acquisition; writing—review & editing; supervision.

## CONFLICT OF INTEREST STATEMENT

The authors declare that they have no known competing financial interests or personal relationships that could have appeared to influence the work reported in this paper.

## Supporting information


**DATA S1:** Supporting Information.

## Data Availability

Data are available upon request.
