# Supplementary material for: Global analysis of kinetics reveals the role of secondary nucleation in recombinant spider silk self‐assembly
Source: Protein Sci. 2023 Aug 1;32(8):e4722. doi: 10.1002/pro.4722 (PMC10364585; doi:10.1002/pro.4722)
Supplement: Supplementary file 1 — DATA S1: Supporting Information. [file PRO-32-e4722-s001.pdf]

Supporting information

for

Global analysis of kinetics reveals the role of secondary nucleation in recombinant spider silk self-assembly

SUPPORTING PROCEDURES

Curve fitting using AmyloFit.

The online platform AmyloFit (www.amylofit.ch.cam.ac.uk) has been used for the global fitting of the kinetic data with the chosen model of secondary nucleation. The entire analysis was performed according to the published protocol (2). Briefly, the normalized kinetic data were uploaded to AmyloFit, which generated the half-times of eADF4(C16) aggregation. The half-time for each curve corresponds to the time point at which the turbidity intensity reaches half of the maximum value between the baseline and the plateau. Half-time values (τ) were then plotted on a double logarithmic plot against the initial monomer concentration of the protein (m_0). The relationship between log(τ) and log(m_0) is described by the equation $\log(\tau) = \gamma\, log\,(m_0) + constant$, where γ is the scaling exponent indicating the slope of double logarithmic plot. A linear slope (power function dependence) shows the independence of scaling exponent on monomer concentration. Deviations of the points from a straight line suggest changes in the dominant mechanism of aggregation with varying monomer concentration. A negative curvature shows a concave plot, i.e., the slope becomes steeper at higher concentrations of protein monomers, which indicates the presence of parallel competitive processes. A positive curvature, on the other hand, implies a convex plot, suggesting a saturation effect. Therefore, the determination of the scaling exponent serves as a valuable first guide for selecting potential models.

In any case, the AmyloFit considers kinetic models that involve nucleation and growth processes, and it describes two quantities: the aggregate mass concentration, $M(t)$, and the aggregate number concentration, $P(t)$. All microscopic processes included in the kinetic models can be categorized into two groups based on whether they affect the aggregate mass or the aggregate number. The three processes considered by the authors of AmyloFit that impact the aggregate number concentration are: (i) primary nucleation, which involves homogeneous

nucleation in solution and is described by a reaction order (n_c) and rate constant (k_n); (ii) secondary nucleation is described by the reaction order (n_2) as well as the rate constant (k_2), and an example is surface-catalyzed nucleation where new aggregates nucleate on the surface of existing aggregates; and (iii) fragmentation, which depends only on the concentration of aggregate mass and has reaction order 1 and a rate constant (k_-). In all cases, the rate constant for elongation (k_+) is considered.

One of the strengths of this platform is the capability of global fitting, also known as shared parameter fitting. This means that large datasets can be fitted simultaneously, and certain parameters such as rate constants and reaction orders can be shared among the datasets. Finally, it is important to validate the selected model through additional experiments, such as the change of the initial fibril concentration, which adds a new degree of freedom that the model has to reproduce correctly, or the addition of seeds to verify of the model of secondary nucleation. The selected model should satisfyingly fit such additional data.

In the present study, the possible models were selected based on the linear dependence of the double logarithmic plots and the negative scaling exponents of the datasets. The reaction orders for primary (n_c) and secondary nucleation (n_2) were fixed across all datasets, with n_c set to 2, and n_2 to 1. In unseeded datasets the corresponding rate constants (k_+k_n and k_+k_2) and in seeded experiments all three rate constants (k_n, k_+, k_2) were set as global (shared) parameters.

Using TEM images and ImageJ software (3), and considering an average protein density of 1.35 g/cm^3 (1),the average fibril length of the seeds was determined to be 495. Subsequently, the aggregate number concentration, P_0, was calculated using the formula M_0/L (2) , where L represents the average fibril length. This approach was employed to minimize the degrees of freedom in the fitting procedure.

The fit including fibril-catalyzed secondary nucleation, where the surfaces of fibrils catalyze the nucleation of new aggregates from monomeric peptide, describes the entire set of time courses.

The system of secondary nucleation can be mathematically represented by the following equations [see SI-S10 and S11 of (2)]:

$$\frac{dP}{dt} = k_n m(t)^{n_c} + k_2 m(t)^{n_2} + M(t) \quad \text{(Eq. 1)}$$

$$\frac{dM}{dt} = 2m(t)k_+ P(t) \quad \text{(Eq. 2)}$$

The resulting fits and corresponding data curves are shown in Figures 4 and 5. The fitting procedure consistently converged to the same value, therefore no errors for individual rate constants are provided. The obtained standard deviations of the data are represented by the filled area surrounding the respective curves.

SUPPORTING FIGURES

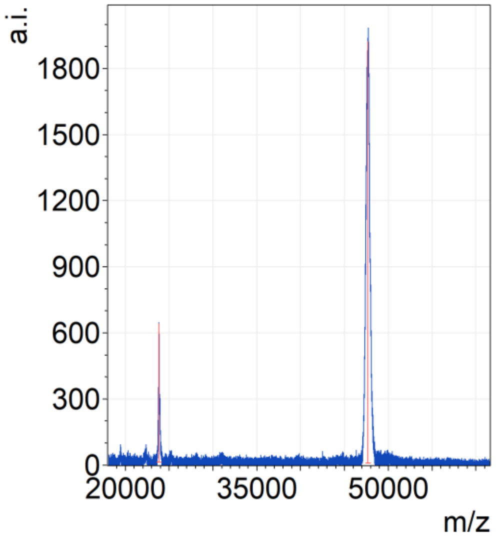

Figure S1. The MALDI-TOF spectrum of the purified protein: found MWs $[M+H]^+$ 47650.9 and $[M+H]^{2+}$ 23816.9, calculated MW: 47698.3 full length, 47567.1 - Met1.

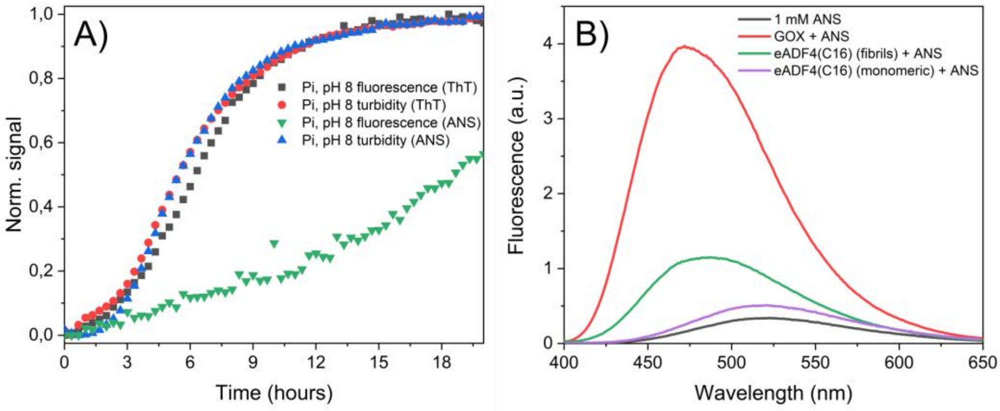

Figure S2. A) Kinetics of self-assembly of 10 μM eADF4(C16) in phosphate buffer (Pi) at pH 8.0, was monitored using fluorescence of 30 μM ThT (ex. at 460 nm, em. at 535 nm) at 30 °C. Simultaneously, change of turbidity of the protein eADF4(C16) was measured in the presence of 30 μM ThT and 1 mM ANS at 340 nm. The kinetics of fluorescence of 1 mM ANS (ex. at 390 nm, em. at 475 nm)in the presence of 10 μM eADF4(C16) was monitored. The ANS fluorescence is normalized to a plateau value of fluorescence reached in ~45 h. B) Fluorescence intensity of (black) 1 mM ANS, (purple) 1mM ANS with the 10 μM protein eADF4(C16) after the ultracentrifugation in phosphate buffer (Pi) at pH 8.0 at the beginning of the reaction, (green) 1mM ANS with 10 μM protein eADF4(C16) that was incubated in phosphate buffer (Pi) at pH 8.0 for 48 hours, (red) 1 mM ANS with termally denaturated 10 μM glucose oxidase from *Aspergillus niger***.** The emission spectra were measured upon excitation at 390 nm.

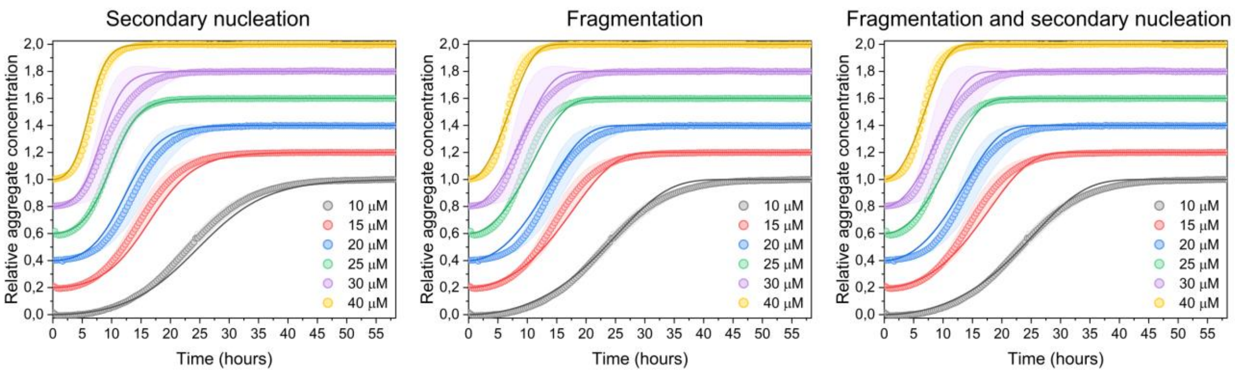

Figure S3. Comparison of fitting results for different aggregation models. The dataset is the same for every model (unseeded experiments at 20°C). For better readability, normalized kinetic data are plotted with constant offset 0.2.

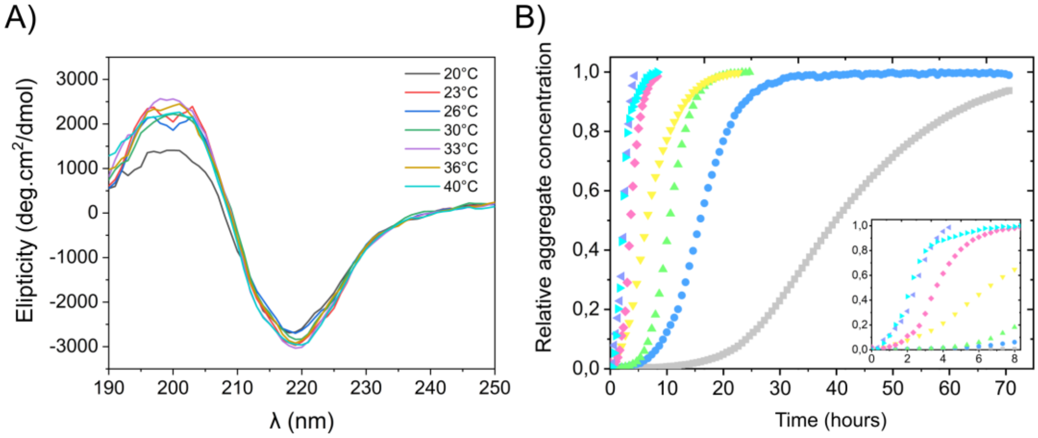

Figure S4. A) The far-UV CD spectra of the protein eADF4(C16) after 48 hours incubation in the presence 150 mM Pi at different temperatures (20-40°C). B) Evolution of turbidity corresponding to fibril formation at 15 µM eADF4(C16) in presence of 150 mM Pi, pH 8.0, at 20°C (grey), 23°C (blue), 26 °C (green), 30 °C (yellow), 33 °C (magenta), 36 °C (purple), 40 °C (cyan). The data points represent an average of turbidity change at 340 nm for 3 replicates for each condition. Inset: kinetic measurements for first 500 minutes. With increasing temperature, an increase in fibrillization rate was observed.

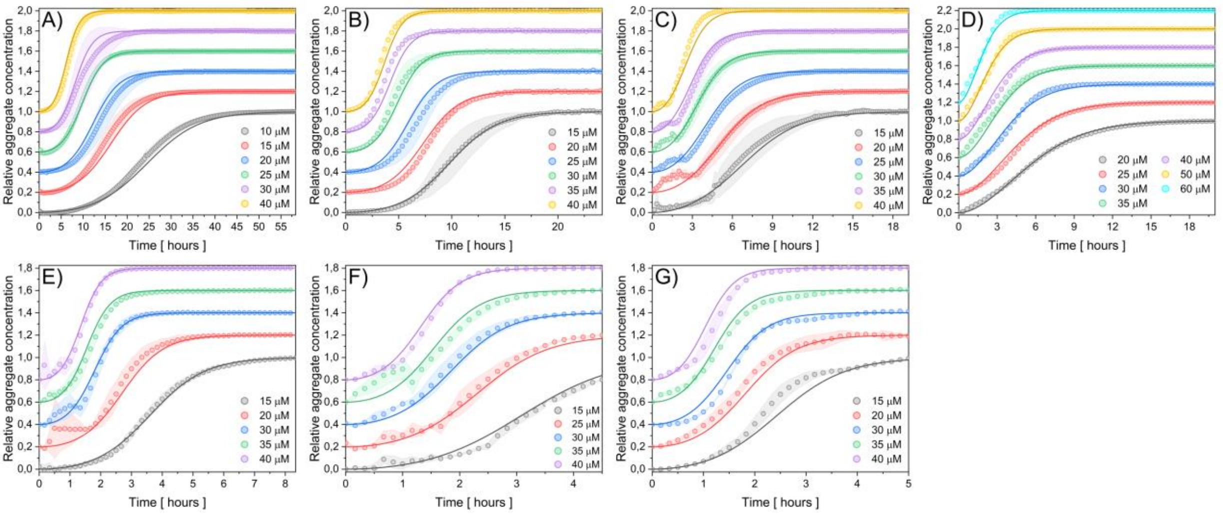

Figure S5. Self-assembly kinetics of eADF4(C16) in the presence of 150 mM KPi, pH 8, at 20°C (A), 23°C (B), 26°C (C), 30°C (D), 33°C (E), 36°C (F), and 40°C (G). Data represent average of 3 replicates for each condition. The global fitting of all data was done using AmyloFit. The best fit was obtained using the secondary nucleation model for all series. The filled area corresponds to standard deviation of the data. In some cases, the standard deviation is smaller than a symbol size. For better visualization, the curves are offset by 0.2 absorbance units. Note the different scales on the time axes.

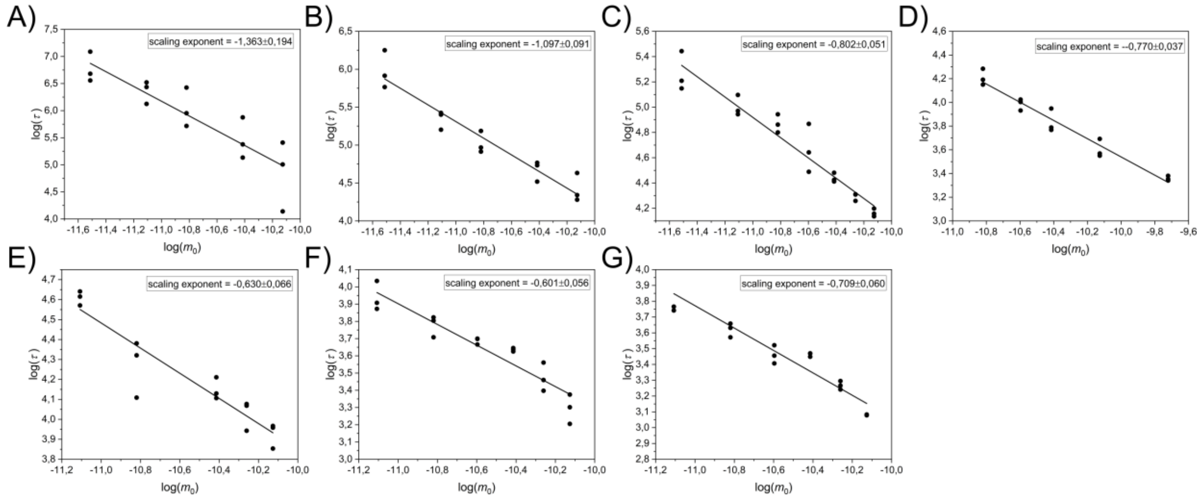

Figure S6. Double logarithmic plots of unseeded reactions - half-times vs. initial protein monomer concentrations for 20°C (A), 23°C (B), 26°C (C), 30°C (D), 33°C (E), 36°C (F), and 40°C (G). Initial protein monomer concentrations were known, and the half-time values were extracted from the corresponding curves shown in Figure 4. The scaling exponents present slope of plots. In all cases, power functions fits indicate that the dominant mechanism does not change at different temperatures.

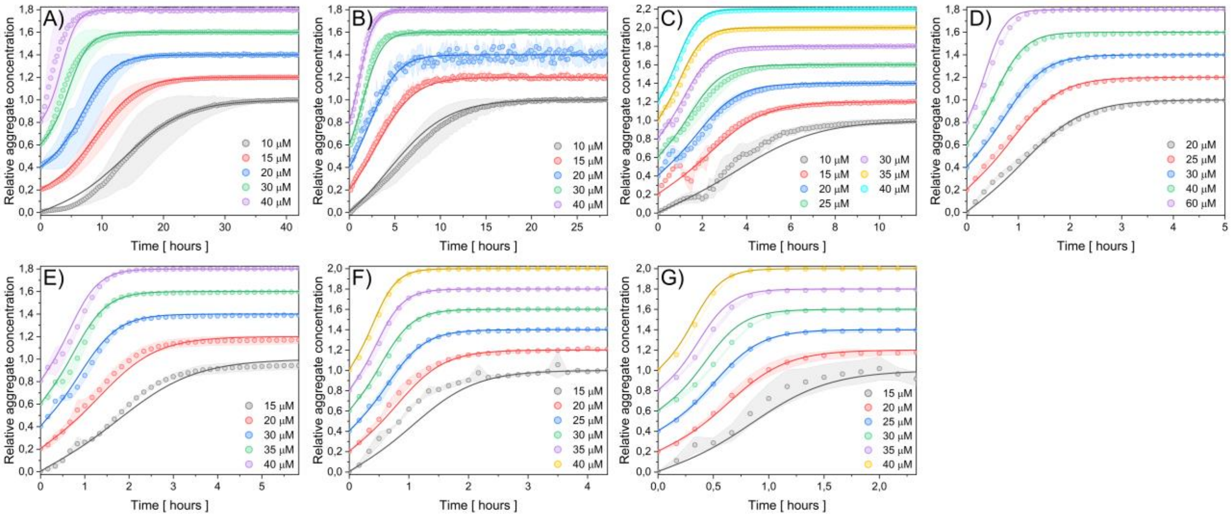

Figure S7. Seeded self-assembly kinetics of eADF4(C16) in the presence of 150 mM KPi, pH 8 and 0.5 % w/w of pre-formed seeds at 20°C (A), 23°C (B), 26°C (C), 30°C (D), 33°C (E), 36°C (F), and 40°C (G). The data represent an average of 3 replicates. The global fitting was performed using the secondary nucleation model. The filled area corresponds to standard deviation of the data. In some cases, the standard deviation is smaller than a symbol size. For better visualization, the curves are offset by 0.2 absorbance units. Note the different scales on the time axes.

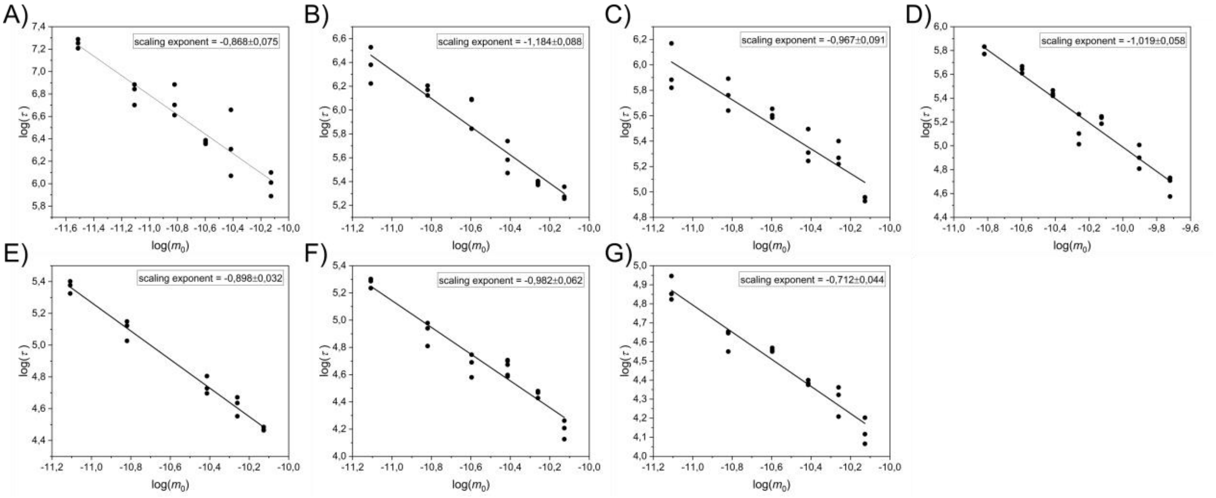

Figure S8. Double logarithmic plots of seeded (0.5% w/w) reactions - half-times and initial protein monomer concentrations for 20°C (A), 23°C (B), 26°C (C), 30°C (D), 33°C (E), 36°C (F), and 40°C (G). Initial protein monomer concentrations were known, and the half-time values were extracted from the corresponding curves shown in Figure 5. The scaling exponents present slope of plots. In all cases, power functions fits indicate that the dominant mechanism does not change at different temperatures.

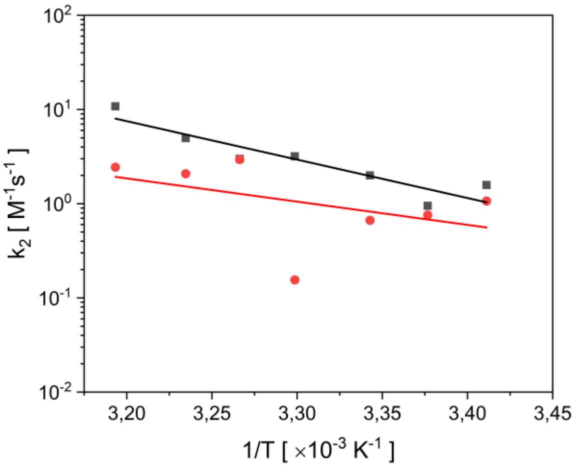

Figure S9. The inverse temperature dependence of k_2 in logarithmic scale. Red points show parameter k_2 obtained by dividing k_+k_2 from unseeded data by k_+ from seeded experiments. Black squares correspond to k_2 obtained from fitting the seeded dataset. Black and red lines represent a linear fit. The observed differences in the parameters k_2, obtained by two different approaches are less than one order of magnitude.

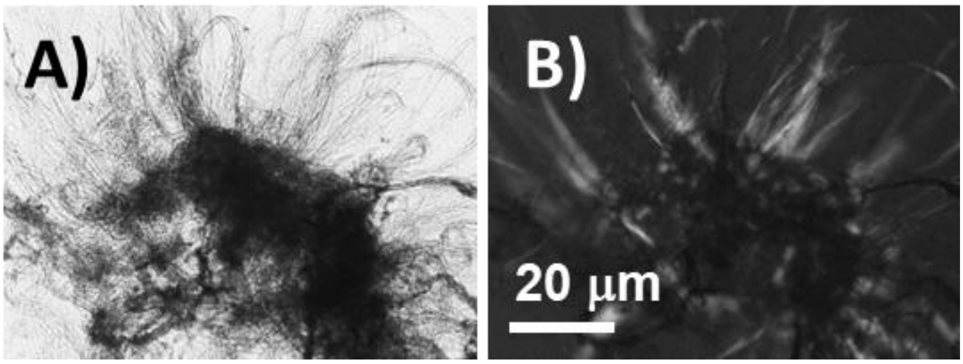

Figure S10. Macroscopic aggregates formed after incubation of eADF4(C16) protein in 150 mM Pi under shear. The aggregates are visualized using (A) bright field microscopy and (B) cross-polarized light microscopy showing birefringence due to fibril's alignment.

References

1. Fischer H, Polikarpov I, Craievich AF (2004) Average protein density is a molecular-weight-dependent function. Protein Science 13:2825-2828. PMID: WOS:000224007700028 {Medline}
2. Meisl G, Kirkegaard JB, Arosio P, Michaels TCT, Vendruscolo M, Dobson CM, Linse S, Knowles TPJ (2016) Molecular mechanisms of protein aggregation from global fitting of kinetic models. Nat Protoc 11:252-272. PMID: WOS:000369084500004 {Medline}
3. Schneider CA, Rasband WS, Eliceiri KW (2012) NIH Image to ImageJ: 25 years of image analysis. Nature Methods 9:671-675. PMID: WOS:000305942200020 {Medline}
